# A note on the history of Hirschsprung’s disease, and an over 120 years apology

**DOI:** 10.1007/s00383-025-06003-z

**Published:** 2025-05-30

**Authors:** Ayşenur Celayir, Handan Çetiner, Canan Aldırmaz Ağartan, Reyhan Günaydın, Arın Celayir

**Affiliations:** 1 Department of Pediatric Surgery, Istanbul Zeynep Kamil Maternity and Children’s Diseases Health Training and Research Center, University of Health Sciences, Istanbul, Turkey; 2 Department of Pathology, Istanbul Zeynep Kamil Women and Children’s Diseases Health Training and Research Center, University of Health Sciences, Istanbul, Turkey; 3 Department of Pediatric Surgery, Retired from University of Onsekiz Mart, Faculty of Medicine, Çanakkale, Turkey; 4 Department of Psychiatry and Psychotherapy, University of Tubingen, Faculty of Medicine, Tubingen, Turkey; 5 Department of Orthopaedics and Traumatology, Istanbul University-Cerrahpaşa, Cerrahpaşa Faculty of Medicine, Istanbul, Turkey

**Keywords:** Hirschsprung disease, Idiopathic colon dilatation, History of medicine, Medical publications, Ganglion cell, K. Tittel

## Abstract

In 1886, Harald Hirschsprung presented what he believed to be a new and rare condition at the Berlin Congress for Children’s Diseases. In 1888, still unaware of earlier reports, he published a paper titled “Constipation in Newborns as a Consequence of Dilation and Hypertrophy of the Colon,” suggesting that the enlargement of the colon was congenital. Although he noted a narrowing of the rectum in his initial case, Hirschsprung attributed this condition to a dilated intestine rather than a narrowed rectum. In fact, F. Ruysch had described a case of over-enlarged colon as early as 1691, and in 1800, D. Battini published another case of severe constipation posthumously. Between 1825 and 1888, around 20 similar cases had been documented in medical literature. K. Tittel was the first person to draw attention to the narrow rectum and the absence or scarcity of ganglion cells in this region in 1901. However, he did not identify this as the exact cause of the disease, This was evident not only in his own article but also in references to his work in other publications. Despite this fact in the literature, the disease is known as “Hirschsprung Disease”. Given the historical and scientific context, it may be time to reconsider the attribution of K. Tittel’s discovery and to recognize the significant contributions of him in understanding of this disease.

## Introduction

The etiology and pathophysiology of Hirschsprung disease is a complex story with a fascinating history regarding its naming. Since the late 1880s, numerous articles have discussed “idiopathic colon dilatation”. These studies are invaluable in defining and attempting to understand the disease’s origins and causes. Harald Hirschsprung believed that congenital dilatation and hypertrophy of the colon were the main causes of the disease, consistently referring to it as congenital dilatation of the colon in his publications [1–4].

**Fig. 1:**
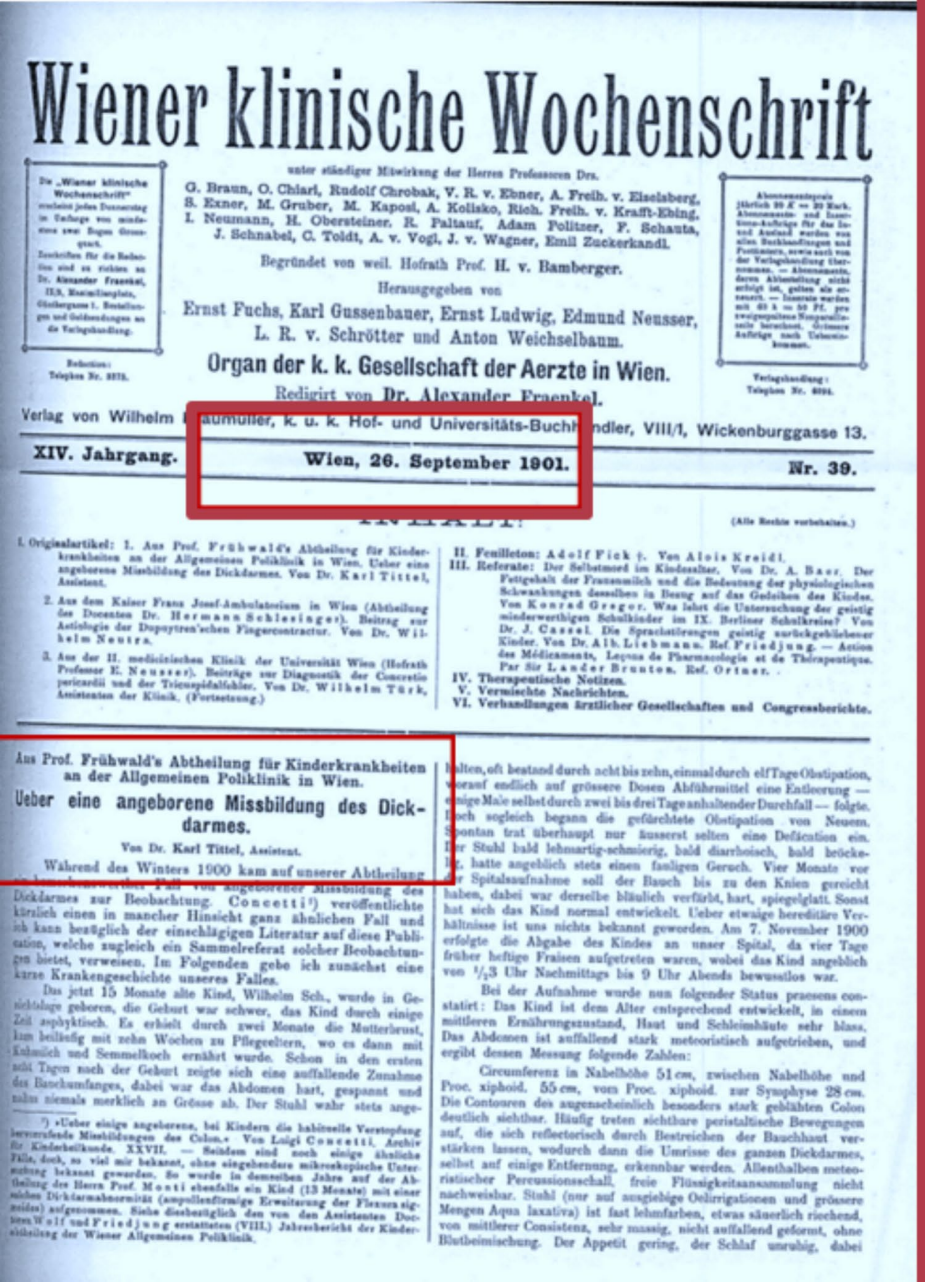
The photocopy of the first page of the Tittel’s article

However, in 1901, Karl Tittel was the first to demonstrate, histologically, the absence of ganglion cells and the thickened, narrowed rectum in these cases, he had published in a weekly journal in Vienna (Fig. 1) [5]. Despite this crucial finding, as early as 1900 and 1904, it was referred to in literature as “Maladie de Hirschsprung” [6], “von Hirschsprungscher Krankheit” and today as Hirschsprung disease [7].

Notably, although Tittel was the first to document a deficiency of rectal ganglion cells in 1901, the name of the disease continues to honor Hirschsprung. This study aims to intend to highlight the forgotten work of Dr. Tittel, to initiate a discussion regarding the naming of Hirschsprung disease, now over 120 years later, by examining relevant literature on its etiopathogenesis in chronological order, ultimately highlighting the inconsistencies and inaccuracies in the naming of this condition.

## Method

All articles on Hirschsprung disease from 1880 to 1950, along with relevant medical historical articles, were thoroughly reviewed. Literature was retrieved via the PubMed system, and full-text versions were accessed in collaboration with the University of Istanbul-Cerrahpaşa and only articles whose full text was available were included in this study. Certain key articles, such as Tittel’s 1901 study, were obtained from the library of the University of Tubingen in Germany.

While extensive information on Dr. Harald Hirschsprung, including details about his family, can be found through internet search engines and PubMed, but Dr. Karl Tittel and his research are not readily accessible through these platforms. Tittel’s study, originally published in 1901 in Wiener Klinische Wochenschrift, is one such example.

Notably, this journal has continued its publication under the same title as “Wiener Klinische Wochenschrift” on PubMed, until today. As Tittel’s article was written in German language in 1901, it was ultimately located in a digital archive of a German university library for this study.

## Results

In 1886, Harald Hirschsprung described what he thought was a new and rare condition at the Berlin Congress for Children’s Diseases. He presented two cases of a 7-month-old and an 11- month-old boy, each with progressive abdominal distention and severe difficulty passing stools, starting at birth were described. At necropsy, the hugely dilated thick walled colon with intraluminal fecalomas, mucosal ulceration and a normal calibered rectum were. In 1888, unaware of previous reports, summarized his findings in an article entitled “Constipation in Newborns as a Consequence of Dilatation and Hypertrophy of the Colon” [1–4].

Whereas before the first description of this disease by Harald Hirschsprung in 1886 (first published in 1888), approximately 20 similar cases had been recorded between 1825 and 1888 years in the literature [1–4, 6–12]. First, a case had been described in 1691 as a phenomenon of an over-enlarged colon by Ruysch [1–4, 8]. The earliest ones were thought to be those of Parry in 1825 and Billard in 1829.” F. Jayle in 1909″ called attention to a report by Frederich Ruysch dated 1691, and now generally accepted as the first recorded instance of this disorder. This was the case of a child who died at the age of 5 years and who was autopsied by Frederick Ruysch. The observation was reported in his work. Observationurn Anatomici-Chirurgicarum Centuria, which was published in Amsterdam in 1691 [8]. A severe case of constipation, which he followed for nearly 10 years, was published as a book in 1800 after Domenico Battini’s death [9]. This book not only provided a very careful account of the patient’s clinical development but also provided a detailed postmortem examination of the abdominal viscera, with reference to changes occurring in the intestines [9]. The unique case reported by Lewitt in 1867 is the first to appear in the American literature [10]. Lewitt’s article, in which he wrote about the postmortem findings of a patient who had had similar attacks repeatedly until the age of 12 and who had not been discharged for 3 weeks, was an important study on colon enlargement. Even, in a 2011 article, the knowledge of ancient Hindu surgeons about Hirschsprung’s disease was demonstrated with evidence from the Sushruta Samhita, dating from around 1200–600 BC [11]. Sushruta Samhita is an ancient tome of Ayurvedic surgery compiled by Sushruta (circa 1200–600 bc). A condition called Baddha Gudodaram in this book, described in the Samhita, closely resembles Hirschsprung disease. There were indications that ancient Indians even deciphered the etiology as defective nerves. Although the ailment was considered incurable, a palliative operation has been discussed as descriptive details of the operation matched with sigmoid colostomy. Evidence from Sushruta Samhita was indicated that Hindu surgeons of prehistoric India probably had considerable knowledge about Hirschsprung disease [11]. While Harald Hirschsprung summarized his findings in a publication in 1888, unaware of previous reports, it can be seen in the literature that this disease was successfully treated years ago [12–14].

In 1893, Walker Griffiths reported that in the autopsy of a child who died with colon dilatation, the longitudinal and circular muscle layer of the hypertrophied colon was hypertrophied and that there was chronic inflammation of the mucous membrane [12]. The rectum was somewhat large for a lad of 11 years, and its muscular walls are thinner than natural. They suggested that this unique condition was initially caused by inflammation of the mucous membrane called colon-colitis and became chronic due to various reasons, that a great intestinal distension occurred due to the changing chemical chains in the stool and that a lot of gas was produced. He also suggested that this expansion of the intestines would naturally make peristalsis more difficult, that the difficulty in pushing the stool forward was compensated by the increase in the size of the muscle fibers and by the formation of new fibers, e.g., a true muscle hypertrophy developed [12].

In an article published in 1898, Treves suggested that cases of “idiopathic colonic dilatation” in children were less “idiopathic” than in the elderly [13]. In this article, in which he presented a young child who clearly showed the features of “idiopathic colonic dilatation”, as indicated by excessive abdominal distension, persistent constipation, hypertrophy of the lower part of the colon, and the practical failure of all purgative measures, he showed that there was evidence that the great majority of these children were due to congenital narrowing of at least the lower extremity of the large intestine.

In 1901, **Karl Tittel** had shown for the first time that there were no ganglion cells in the distal rectum [5]. The case of a 15-month-old infant with abdominal distension was that started 8 days after birth and who suffered from the lack of a bowel movement for 11 days. The child was developing fairly well while on breast feeding. After the child was switched to cow’s milk, his/her health status deteriorated and the child died of bowel obstruction. Once again, an autopsy revealed megacolon, and as Tittel noted, it was very interesting that the ganglia of myenteric plexus could hardly be found. Normal findings were found in ileum. Tittel thought it could be impaired bowel fixation, but he almost immediately doubted this thesis when a well-defined sympathetic ganglion was found outside the rectum wall. More compelling was his notion that a probable cause of impaired peristalsis was not macroscopically detected changes of the bowel but a disorder of intramural innervation [5]**.**

**Fig. 2:**
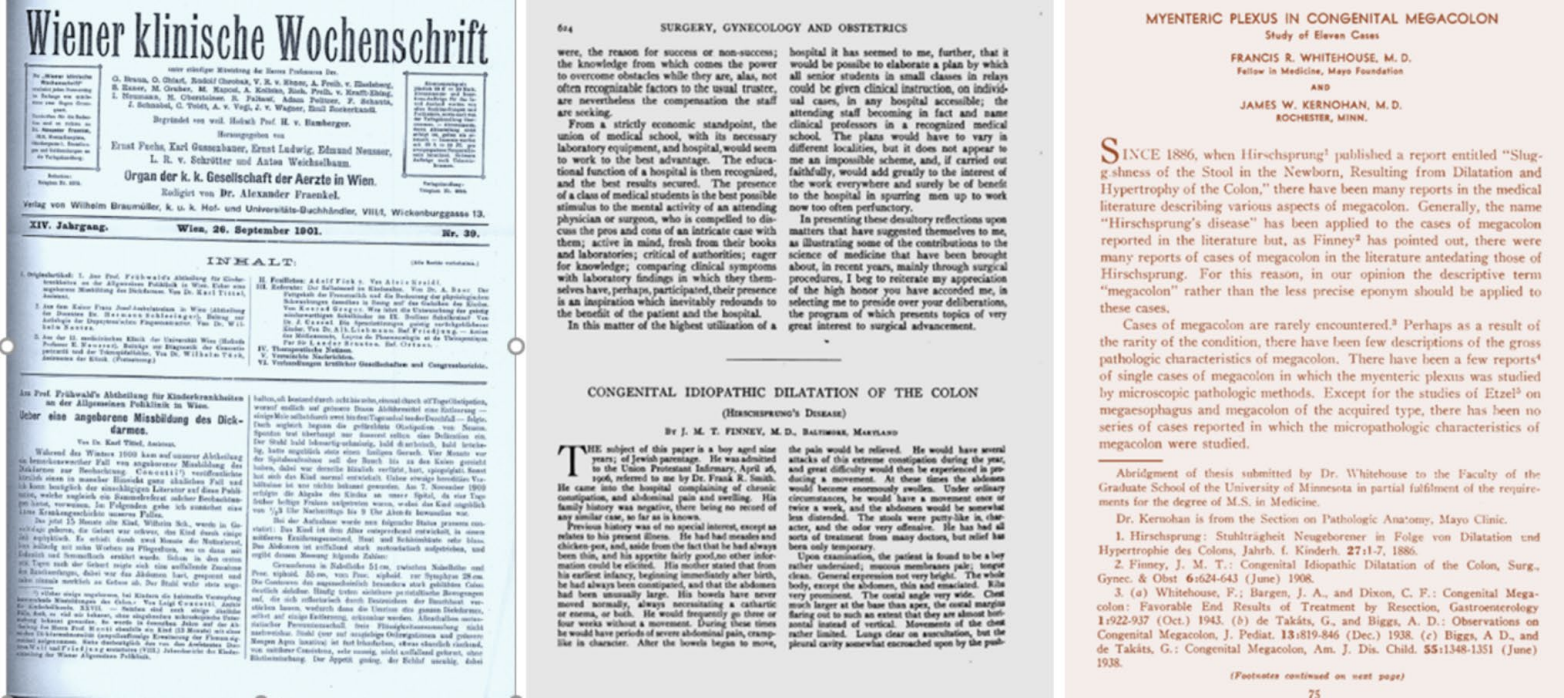
Photocopies of first pages from articles of Tittel’s, Finney’s and Whitehause and Kenohans’ are seen as respectively

There are important articles about this disease in the literatures between 1880 and 1950, especially two systematic reviews by Finney (1908) and Whitehause & Kernohan (1948) among these articles were particularly interesting and informative, as well as the work of Tittel’s study [14, 15] (Fig. 2).

The first of is titled “Congenital Idiopathic Dilatation of the Colon (Surg Gynec&Obst, 1908;6:624–643)” in which 246 references were reviewed and written systematically by JMT Finney [13] (Fig. 2). Finney presented a 9-year-old boy who had constipation and abdominal distension since birth [14]. Findings of Hirschsprung disease were very typical in his history. In the abdominal exploration, it was observed that the diameter of the sigmoid colon, which was severely dilated hypertrophied, reached 14 cm, its mesentery was thickened up to 3 cm, and the other intestines were normal; and the patient underwent a right transverse colostomy. Finney’s study on congenital megacolon is one of the most comprehensive studies on this subject and examined all studies on congenital megacolon as well as the presentation of his own case. In this article, he made a comprehensive review of 246 articles on congenital megacolon (clinical presentation, medical history and naming of the disease, etiological causes, diagnosis, treatment and histopathological evaluations) [14].

One of the highlights of Finney’s article was naming of the disease [14]. It has been reported that the disease had been called as Hirschsprung disease by European authors since 1900, but it has been named as congenital colonic dilatation in non-European countries [1, 2, 4, 6–14]. In Finney’s article, he also mentioned Hirschsprung disease from page from especially where the authors he cited discussed their topics [14]. Although it has been determined that “German and Dutch authors used it as Hirschsprung’s Disease, while American, English and French used it as “congenital idiopathic dilatation of the colon”, but Finney it used as “Hirschsprung Disease” from on page 628 of his study [14].

**Fig. 3:**
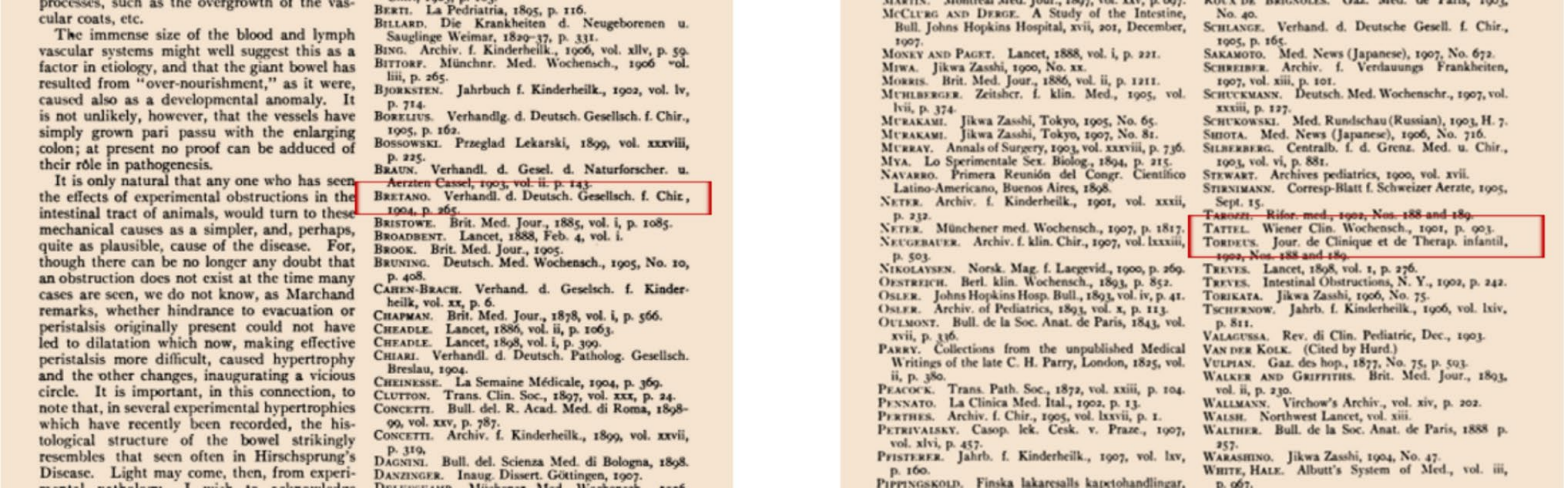
In the references list of Finney’s article, it is seen that both Tittel and Brentano were miswritten with a letter error as TATTEL and BRETANO

On page 640 of Finney’s article*,* “*Indeed, there has been repeated suggestion of congenital absence of the normal nervous plexus in the colon wall*.” is written, but which author’s work is not mentioned [14]*.* Tittel (1901), and Brentano (1904), who reported the absence or scarcity of ganglion cells in the rectum in two studies until 1908, appear to be included in the references section of Finney’s article. But, both authors’ names were miswritten with a letter error in the form of Tattel and Bretano by Finney (Fig. 3).

**Fig. 4:**
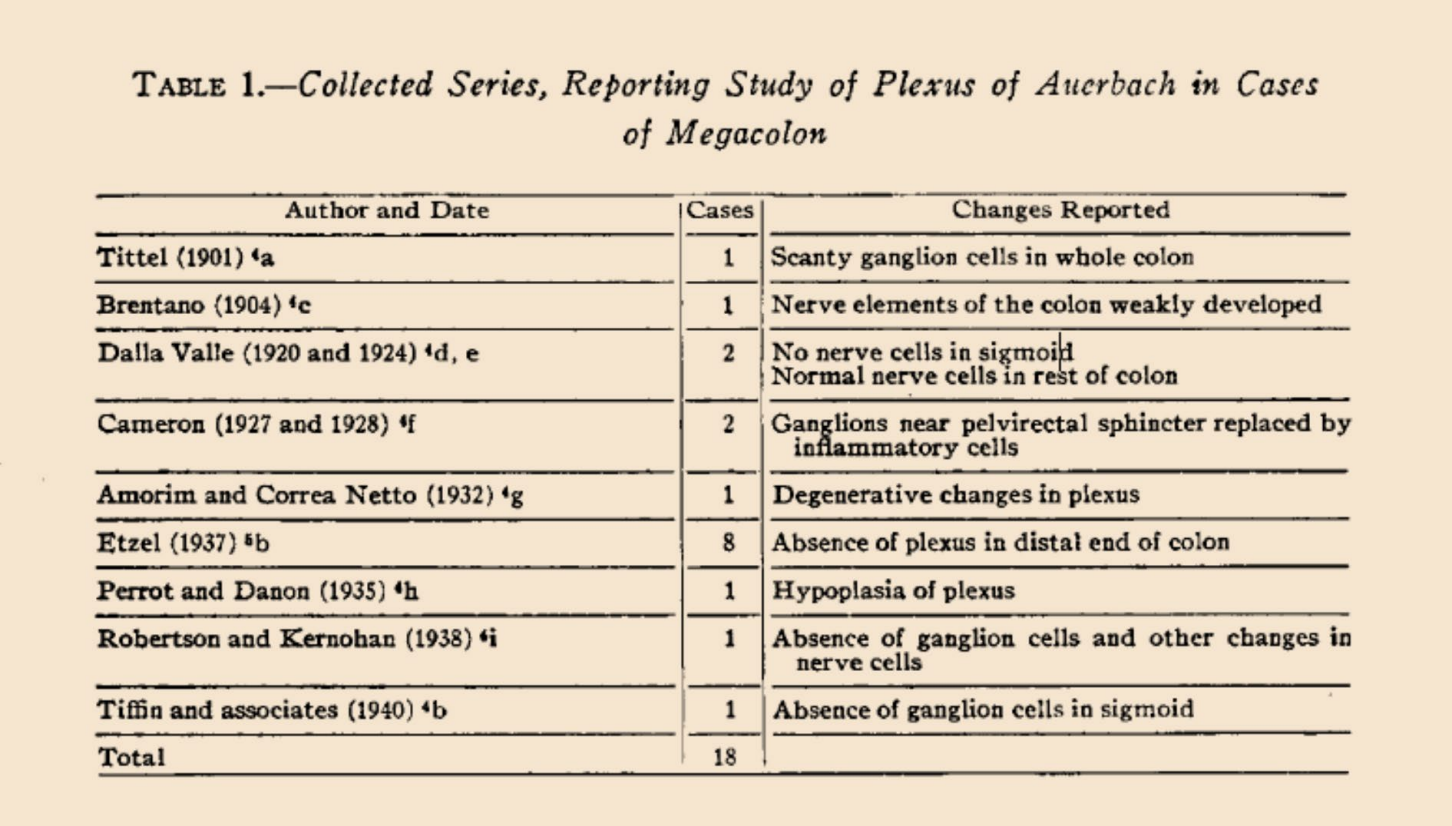
In this table of Whitehause and Kerhonan’s article [18] is seen that in 14 of 18 cases collected from the literature, there was absence of the ganglions of the myenteric plexus in the distal part of the colon or in a part of the colon not specified. The ganglions of the myenteric plexus in the distal part of the colon or in a part of the colon not specified were absent

The second important study in the Literature is titled “Myenteric Plexus in Congenital Megacolon: Study of Eleven Cases” by Whitehouse FR, and Kernohan JW [15] (Fig. 4). In that study, the presentation of their 11 cases with congenital megacolon and literature review about congenital megacolon histopathology were made. Other ten authors data were collected from the literature, 14 of these 18 cases showed that myenteric plexus ganglia were not found in the distal part of the colon or in an unspecified part of the colon. Those studies were listed in chronological order in a table in that study by Whitehouse FR and Kernohan JW. In this histopathological review, study made in 1948, and Tittel (in 1901) was shown in the first place and Brentano (in 1904) was shown in the second place in a table (Fig. 4). Both authors reported scarcity or absence of ganglion cells in their study [15].

The literature on congenital aganglionic megacolon discussed in this study is listed separately in chronological order in Table 1 (Table 1).

**Table 1:** Chronological order of leading articles. The table is marked with an (*) by authors who indicated that there were no ganglion cells in the rectum and colon

Year	Author	Title of article	Medical definition used diagnosis and clinical definition		Language of the article
1691	F. Ruysch	Leenders E, Sieber WK. Congenital megacolon observation by Frederick Ruysch-1691. J Pediatr Surg 1970;5(1):1–3	Enormous dilatation of the colon	Autopsy	Dutch
1867	W. Lewitt	Lewitt W. Enlargement of the colon. Chicago Med J 1867;24:359	Enlargement of the colon	Clinic	English
1886–1888	H. Hirschsprung	Hirschsprung H. Stuhlträgheit Neugeborener in Folge von Dilatation und Hypertrophie des Colons. Jahrbuch für Kinderheilkunde und physische Erziehung 1888; 27: 1–7	Colonic dilatation and hypertrophy	Clinic	German
1893	TJ Walker, J Griffiths	Walker TJ, Griffiths J. Congenital (?) dilatation and hypertrophy of colon fatal at age of 11 years. Br Med J 1893;2:230–231	Congenital (?) dilatation and hypertrophy	Clinic + autopsy, Histopathologic: [muscle hypertrophy]	English
1898	F. Treves	Treves F. Idiopathic dilatation of the colon. Lancet 1898;29:276 -279	İdiopathic dilation of the colon	Clinic	English
1900	KG Lennander	Lennander KG. Fall af medfiidd (?) dilatation och hypertrofi af flexura sigmoidea hos eft barn (maladie de Hirschsprung). Nord Med Ark 1900;2:1	Colon hypertrophy/Hirschsprung’s diseases	Clinic, autopsy	Sweden
1901	*K Tittel	Ueber eine angeborene Missbildung des Dickdarmes, Wien Klin Wchnschr 1901:14:903–907	Rectal aganglionosis	Histopathologic	German
1904	A Brentano	Brentano A. Uber einen Fall von Hirschsprungscher Krankheit. Verh Dtsch Ges Chir 1904;1:265–268	Rectal aganglionosis	Histopathologic	German
1908	JMT Finney	Finney JMT. Congenital Idiopathic Dilatation of the Colon. Surg Gynec & Obst, 1908;6:624–643	Congenital idiopathic dilatation	Clinic, review	English
1924	A Dalla Valla	Dalla Valla A. Contributo alla conoscenza della forme famigliare del megacolon congenito. Pediatria 1924;32:569–599	Congenital megacolon	Histopathologic	Portuguese
1927	JAM Cameron	Cameron JAM. Oesophagectasia in Child. Arch Dis Childhood 1927;2:358–360	Congenital megacolon	Histopathologic	English
1938	HE Robertson, JW Kernohan	Robertson HE, Kernohan JW. The myenteric plexus in congenital megacolon. Proc Mayo Clin 1938;13:123–125	Congenital megacolon	Histopathologic	English
1940	ME Tiffin	Tiffin ME, Chandler LR, Faber HK. Localized absence of ganglion cells of myenteric plexus in congenital megacolon. Am J Dis Child 1940;59:1071–1082	Congenital megacolon	Histopathologic	English
1948	FR Whitehouse, JW Kernohan	Whitehouse FR, Kernohan JW. Myenteric Plexus in Congenital Megacolon: Study of Eleven Cases. Arch Intern Med (Chic) 1948;82(1):75–111	Congenital megacolon	Histopathologic	English
1955	O Swenson, JH Fisher, HE MacMahon	Swenson 0, Fisher JH, MacMahon HE: Rectal biopsy as an aid in the diagnosis of Hirschsprung’s disease. N Engl J Med 1955;253:632–635	Distal colon aganglionosis Hirschsprung’s disease	Histopathologic	English
1973	O Swenson, JO Sherman, JH Fischer	Swenson O, Sherman JO, Fischer JH. Diagnosis of congenital megacolon: an analysis of 501 patients. J Pediatr Surg 1973;8(5):587–93	Congenital megacolon	Clinic and histopathologic	English

*The earliest description of the term “rectal aganglionosis”

## Discussion

A historical review of clinical diseases is important for several reasons. Historical events reveal how uncertainties in clinical diseases often resolve gradually. The discovery, naming, and pathophysiology of Hirschsprung’s disease become more evident when studies on the subject are analyzed in a historical and chronological order. [1–4, 6–14].

The first histological report on ganglion cells in Hirschsprung disease was by Tittel in 1901 [5]. The intrinsic plexus was sparsely developed throughout the colon, but was normal in the ileum. Tittel also stated that the rectum was narrow in his case [5]. However, he was not yet aware that the underlying cause of the disease was aganglionosis. It is not possible for him to say this with a case report anyway in that years. In 1904, Brentano reported similar findings [7].

The review of Finney [14], who systematically wrote down all the known information about congenital idiopathic dilatation of the colon with the information of those days by reviewing of 246 articles in 1908, is a very important study. Finney cited Tittel’s study, which showed the absence of ganglion cells [14]. He mentioned three theories about Hirschsprung disease in his study; however, Finney’s unawareness of the importance of the discovery of aganglionosis by Tittel due to a single case is an acceptable situation under the circumstances of those days. Likewise, Finney did not realize the importance of Tittel’s discovery because it was the only case [5, 14]. Brentano’s cases were also not sufficient to understand the importance of aganglionosis at that time [7, 14]. However, in Finney’s article, although the article title and journal names are written correctly in the references section, it is seen that both authors’ surnames are misspelled with a letter error (in the form of Tattel and Bretano instead of Tittel and Brentano) [14]. However, when the original articles are examined, it is understood that the author’s names are written incorrectly in the source of this article. In fact, after the 1900s, the nomenclature of Hirschsprung disease was started to be used in the literature by Dutch authors [7, 9, 14].

Total absence of ganglion cells was described in 1920, 1924 by Dalla Valla [16], in 1928 by Cameron [17], in 1938 by Robertson and Kernohan [18], and in 1940 by Tiffin et al. [19]. But all these were isolated reports and as mentioned previously, other workers had reported normal histological findings. Aganglionosis had, therefore, been thought to represent a rare secondary event. The 1948 article by Whitehouse FR and Kernohan JW left no doubt that the ganglion cells were absent [14, 18]. What a great misfortune that the importance of these two authors (Tittel and Brentano) and their articles were only understood and appreciated in by Whitehouse FR and Kernohan JW’s article in 1948 [5, 7, 18]. After the pathophysiological characterization of HD in 1948 by Whitehouse and Kernohan, rectal biopsy became the method of choice for confirming the diagnosis. In 1955, Swenson demonstrated the possibility of performing the diagnosis with 98% accuracy in fragments of the total rectal wall using hematoxylin and eosin staining, since the identification of neurons in the myenteric plexuses rules out the diagnosis [20, 21].

Finally, the disease was discovered to be caused by aganglionosis, and it was also shown in the 1948 study by Whitehouse and Kernohan that Tittel was the first author to show rectal aganglionosis [1, 2, 4, 18, 20–22]. As can be seen from Table 1, although the absence of ganglion cells in the narrowed rectum was first shown in 1901, and Tittel was also cited in the references in Finney’s article in 1908, and despite the fact that distal colon aganglionosis was shown by Tittel to be the first author to define the main fact as aganglionosis in Whitehouse & Kernohan’s 1948 study, a major turnabout in the understanding of the pathogenesis and treatment of Hirschsprung disease came in 1948. It is also acceptable that it was referred to as Hirschsprung disease until 1948. But even after the etiopathogenesis was clearly elucidated, it continued to be called Hirschsprung disease.

With the opportunities available at the beginning of the twentieth century, it was not possible for the authors to access all the literature. With today’s technology, it is possible to access almost all of literature in the twenty-first century. Unlike 1888 or 1901, in the age of digitalization, the information needed is more easily and accurately accessed. Therefore, it is possible to appreciate Tittel’s discovery of aganglionosis through a real re-evaluation based on concrete contributions to the medical literature.

Karl Tittel was the first person to draw attention to the narrow rectum and the absence and scarcity of ganglion cells in this region. This was clearly seen not only in his own article but also in his references in other articles. But he did not reveal this as the exact cause of the disease. Despite this fact, it seems that this important discovery has not been taken into consideration in the literature for over 120 years.

In conclusion, after more than 120 years, we know it is hard to consider renaming well-known “Hirschsprung Disease” and to reflect who first identified its underlying pathology more accurately. However, it would be scientifically appropriate to acknowledge the contributions of the individual who first demonstrated rectal aganglionosis and deserves recognition. Otherwise, Karl Tittel and his contribution will vanish from the historical narrative of Hirschsprung disease.

## Data Availability

No datasets were generated or analysed during the current study.
